# Pragmatic Language Skills: A Comparison of Children With Cochlear Implants and Children Without Hearing Loss

**DOI:** 10.3389/fpsyg.2019.02243

**Published:** 2019-10-09

**Authors:** Michaela Socher, Björn Lyxell, Rachel Ellis, Malin Gärskog, Ingrid Hedström, Malin Wass

**Affiliations:** ^1^Swedish Institute of Disability Research, Linköping University, Linköping, Sweden; ^2^Special Needs Education, University of Oslo, Oslo, Norway; ^3^Department of Clinical and Experimental Medicine, Linköping University, Linköping, Sweden; ^4^Department of Business Administration, Technology and Social Sciences, Luleå University of Technology, Luleå, Sweden

**Keywords:** pragmatic language ability, hearing loss, cochlear implant, verbal fluency, children

## Abstract

Pragmatic language ability refers to the ability to use language in a social context. It has been found to be correlated with success in general education for deaf and hard of hearing children. It is therefore of great importance to study why deaf and hard of hearing children often perform more poorly than their hearing peers on tests measuring pragmatic language ability. In the current study the Pragmatics Profile questionnaire from the CELF-IV battery was used to measure pragmatic language ability in children using cochlear implants (*N* = 14) and children without a hearing loss (*N* = 34). No significant difference was found between the children with cochlear implants (CI) and the children without hearing loss (HL) for the sum score of the pragmatics language measure. However, 35.71% of the children with CI performed below age norm, while only 5.89% of the children without HL performed below age norm. In addition, when dividing the sum score into three sub-measures: Rituals and Conversational skills (RCS), Asking for, Giving, and Responding to Information (AGRI), and Nonverbal Communication skills (NCS), significant differences between the groups were found for the NCS measure and a tendency for a difference was found for the RCS measure. In addition, all three sub-measures (NCS, AGRI, RCS) were correlated to verbal fluency in the children with CI, but not the children without HL.

## 1. Introduction

Pragmatic language ability refers to the ability to use language in a social context. It has been shown to be related to core language ability, including language comprehension and vocabulary skills, and also to cognitive skills (Matthews et al., 2018) for example inhibition, shifting, working memory (Channon and Watts, 2003; Blain-Briére et al., 2014) and reasoning ability (Turkstra et al., 1996). Children with autism spectrum disorder (Norbury and Bishop, 2002; Volden et al., 2009), children with ADHD (Camarata and Gibson, 1999; Kim and Kaiser, 2000; Staikova et al., 2013), and deaf and hard of hearing children (Jeanes, 2000; Most et al., 2010; Goberis et al., 2012; Rinaldi et al., 2013) tend to show poorer performance on several pragmatic language measures compared to typically developing children. Pragmatic language ability seems to be associated with success in general education for deaf and hard of hearing children (Thagard et al., 2011). Thagard et al. (2011) showed that children with higher pragmatic language ability also have higher scores on tests measuring preparedness for first-grade work, math, and reading. Furthermore these children spend more time in general education settings. However, the causal direction of the relationship is unclear. Other studies have suggested that pragmatic language ability is less developed in deaf and hard of hearing children as both the quality and quantity of their daily face-to-face discourses are reduced (Jeanes, 2000; Most et al., 2010). Most et al. (2010) argue that a delay in language development resulting in less flexible use of language structures, reduced audibility during interactions and difficulties with theory of mind might be reasons for the differences seen between children with normal hearing and deaf and hard of hearing children. However, only few and quite diverse studies have focused on children with cochlear implants (CI) and their pragmatic language ability (Jeanes, 2000; Toe et al., 2007; Most et al., 2010; Thagard et al., 2011; Dammeyer, 2012; Goberis et al., 2012; Rinaldi et al., 2013; Toe and Paatsch, 2013).

Pragmatic language skills is an umbrella term for a number of complex verbal and non-verbal skills needed for real-life conversations. These skills range from responding to utterances in an appropriate way, maintaining the topic of the conversation, initiating new, and relevant topics (Matthews, 2014), to not inappropriately interrupt the other speaker, turn-taking (Bonifacio et al., 2007; Longobardi et al., 2017), the ability to ask for clarification and adapting the language to the needs of the conversational partner (Longobardi et al., 2017). In order to be able to successfully use these skills it is important to be able to consider all or some of the following: the context of an utterance (Loukusa et al., 2007; Matthews et al., 2018), acoustic cues like intonation and stress (Paradis, 1998; Most et al., 2010), and non-verbal cues (Russell and Grizzle, 2008). Pragmatic language skills develop during childhood (Loukusa et al., 2007; Longobardi et al., 2017). Mastering these complex skills takes until adolescence or even early adulthood (Matthews, 2014). Pragmatic language skills have been linked to social competence (Conti-Ramsden and Botting, 2004), peer relationship, mental health (Helland et al., 2014), and collaborative-based learning (Murphy et al., 2014).

Children with CI have been found to perform more poorly on a number of pragmatic language abilities. Jeanes (2000) analyzed referential communication between children (four age groups: 8-, 11-, 14-, and 17-year old) and found that profoundly deaf high school students using oral communication use requests for clarification more often than their hearing peers. However, in comparison to the hearing group, the requests were more often nonspecific, which led Jeanes (2000) to suggest that the communicative competence of the deaf and hard of hearing children is not as mature. Ibertsson et al. (2009) as well found teenagers with CI to use more requests for clarification when communicating with a well-known peer without a hearing loss (HL). However, in contrast to Jeanes (2000) in the study by Ibertsson et al. (2009) the teenagers with CI mostly used specific requests for clarifications. Ibertsson et al. (2009) argue that this is a way to control the conversation more. In accordance to this a more recent study done by Toe and Paatsch (2013) indicates that the pragmatic language skills of children with CI at age 9–12 are good enough to ensure a fluent conversation, but that they tend to control the conversation more than children without HL. Toe and Paatsch (2013) analyzed 10 min spontaneous conversations between children with CI and children without HL the same age. Toe and Paatsch (2013) suggest that children with CI try to control the conversation more in order to prevent conversation breakdown. In addition, the results found by Toe and Paatsch (2013) indicate that children with CI have problems with contingency, the ability to maintain the topic of the conversation and to add new and relevant information. This is in accordance with results found by Most et al. (2010). The authors evaluated spontaneous communication between children age 6 and 9 and a familiar adult. Most et al. (2010) found that both children with CI and children with hearing aids (HA) showed problems in the area of turn taking, the ability to have a conversation with smooth interchanges between the conversational partners. This was especially the case for contingency, a skill which none of the children with CI or HA used appropriately, and response and adjacency (no pause between the utterance of the conversational partner and the child's utterance), two skills which were only used appropriately by two of the children with CI or HA. In the studies by Jeanes (2000), Most et al. (2010), and Toe and Paatsch (2013) one instance of conversation in the lab was analyzed. One disadvantage with this approach is that it is not clear whether results translate to real-life, where children need to communicate with different partners in different settings. In order to capture how well children are doing in real-life, other studies have used questionnaires to measure pragmatic language skills in children with CI. Goberis et al. (2012) used a checklist with items covering six categories: states needs, gives commands, personal interaction, wants explanation, shares knowledge, and shares imagination. Parents were then asked to rate a number of skills in each category to be: not present, preverbal, uses on to three words, or uses more complex language. By age six children with CI only used complex language for 6.6% of the items and by age seven they used complex language for 69% of the items. In comparison, children without HL used complex language for 100% of the items by the time they were 6 years old. In contrast to that, results from Guerzoni et al. (2016) suggest that already toddlers with a CI have pragmatic language skills comparable to hearing toddlers. Guerzoni et al. (2016) used a questionnaire using two scales, one for assertiveness and one for responsiveness. The assertiveness scale included items covering the ability to ask questions, make requests, and make suggestions, while the responsiveness scale covered the ability to respond to questions and requests, and turn taking. However, in contrast to the study by Goberis et al. (2012) parents only rated how often a certain behavior occurred. As the children in the study by Guerzoni et al. (2016) were only around 2 years of age it might be that differences between the groups were not apparent because they are only observed for more complex skills and more complex conversations, which a toddler might not yet have. Overall it seems like children with CI have problems with some but not all domains of pragmatic language ability. It should be emphasized that there are only very few studies studying pragmatic language ability in deaf and hard of hearing children and those existing are very diverse, using different age groups and measures. In addition, there is a large time gap between some of the studies. It is therefore unclear if technical improvement of cochlear implants, changes in rehabilitation programs, the use of different measures or the age of the participants have led to different results. The present study aims to get an insight into the current real-life pragmatic language skills of children with CI and to compare them to those of children without HL.

It has been suggested that pragmatic language ability is not only connected to other language skills but also to different cognitive abilities (Turkstra et al., 1996; Channon and Watts, 2003; Martin and McDonald, 2003; Douglas, 2010; Blain-Briére et al., 2014; Matthews et al., 2018). Matthews et al. (2018) point out that it is hard to distinguish between pragmatic language ability and the ability to understand words and sentences. The authors add that some children still mainly show language problems in a social context and that it is therefore important to try to separate these skills. It is not surprising that most studies reviewed by Matthews et al. (2018) found correlations between “formal language” (vocabulary and grammar) and pragmatic language ability. The ability to understand sentences and words do not, however, seem to be the only important skills. Other studies have also shown associations to reasoning ability (Turkstra et al., 1996), cognitive flexibility (Ketelaars et al., 2012; Bacso and Nilsen, 2017), working memory, inhibition, and shifting ability (Channon and Watts, 2003; Blain-Briére et al., 2014; Matthews et al., 2018). Children with CI have been found to perform more poorly than children without HL on a number of executive function skills, like working memory (Wass et al., 2008; Kronenberger et al., 2013), reasoning (Bandurski and Ga1kowski, 2004; Edwards et al., 2011), and cognitive flexibility (Kenett et al., 2013; Wechsler-Kashi et al., 2014). These abilities seem to be associated with pragmatic language ability in normally developing children (Turkstra et al., 1996; Ketelaars et al., 2012; Blain-Briére et al., 2014; Bacso and Nilsen, 2017; Matthews et al., 2018). A delay in these cognitive functions might therefore lead to a delay in pragmatic language skills. However, the association between these cognitive skills and pragmatic language ability in children with CI has not yet been studied. Previous research suggests that the development of certain pragmatic language skills is delayed in children with CI compared to children without HL even when being matched on language ability (Most et al., 2010). This indicates that other factors apart from language ability play a role. To our knowledge there is no study looking into the connection between language measures, cognitive measures and pragmatic language ability in children with CI in comparison to children without HL. This study aims to take the first step in filling this research gap.

## 2. Methods

### 2.1. Participants

Fifty-five children participated in the study. Seventeen of them were fitted with cochlear implants (CI). The 17 children with CI were recruited from a special school as well as via the hearing clinic in Stockholm, Sweden. They attended pre-school class, first grade, and second grade, respectively. The hearing loss of one child was caused by Usher syndrome. This syndrome leads also to a visual disability. Unfortunately no data concerning the visual impairment was collected. However, it was not reported by the test leader that any visual problems occurred during testing. To our knowledge, none of the other children had any additional disability apart from their hearing loss. Three of the children with CI were excluded from the study. One because the parents did not fill in the Pragmatics Profile and two as data on three of the other measures were missing. The mean age of the remaining 14 children (10 girls) was 6.77 years with a standard deviation of 11.13 months. Three of the children were unilaterally implanted and 11 had bilateral CIs. Their deafness was detected at a mean age of 11.14 months, with a standard deviation of 13.84 months. They received their implants at a mean age of 24.07 months with a standard deviation of 19.55 months. Two of the children were bilingual (using sign language and oral language). Four children used only oral language. The remaining eight children used oral language as their main communication mode and signs for support. One of those eight children was reported to not sign him/herself, but the parents used signs as support. A detailed description of the children with CI is provided in Table 1. The 38 children without HL were recruited from schools in Linköping, Sweden and attended a pre-school class. Four of the children without HL were excluded from the analysis. One because the test session was interrupted several times, one because s/he was not able/willing to finish all tasks and two because the parents did not fill in the Pragmatics Profile. The mean age of the remaining 34 children (17 girls) was 6.52 years with a standard deviation of 4.01 months. Thirty of the children took part in an intervention study and the results reported here are their pre-test results. The children without HL were tested individually, either during school time in a separate room or at home. The children received stickers for their participation. A consent form was signed by the caregivers. Both children and caregivers were told that they could drop out of the study at any point without giving a reason. The study was approved by the Linköping Research Ethics Committee (dnr 2015/308-31).

**Table 1:** Individual data – Children with CI: The data were collected using a questionnaire which was filled in by the caregivers.

**Age**	**Detection of deafness**	**First implant (CI)**	**Unilateral/Bilateral**	**Communication mode**	**Schooling**
8 years 9 months	2 days	24 months	Bilateral (30 months old)	Oral (10% sign)	Special
8 years 7 months	12 months	24 months	Bilateral	Oral (10% sign)	Special
8 years 11.5 months	44 months	60 months	Unilateral	Only oral	Special
8 years 4 months	30 months	36 months	Bilateral	Only oral	Special
7 years 9 months	Newborn	12 months	Bilateral (18 months old)	Oral (sign support)	Special
7 years 4 months	1 month	66 months	Unilateral	Oral (10% sign)	Special
6 years 6 months	2 months	8 months	Bilateral (20 months old)	Bilingual (Oral + sign)	Special
6 years 3 months	newborn	36 months	Unilateral	Bilingual (oral + sign)	Special
5 years 8 months	3 months	7 months	Bilateral (12 months old)	Only oral	Mainstream
5 years 9 months	1 month	8 months	Bilateral (18 months old)	Only oral	Mainstream
7 years	6 months	12 months	Bilateral (20 months old)	Oral (sign as support; not signing self)	Mainstream
5 years 8 months	3 months	7 months	Bilateral (15 months old)	Oral (sign as support)	Mainstream
5 years 7 months	24 months	28 months	Bilateral (38 months old)	Oral (sign as support)	Mainstream
7 years	6 months	9 months	Bilateral (11 months old)	Oral (sign as support)	Mainstream

### 2.2. Material

#### 2.2.1. Pragmatic Language Ability

The pragmatic language ability of the children was tested using the Pragmatics Profile from the Swedish version of the clinical evaluation of language fundamentals 4 screening test battery—CELF-IV (Semel et al., 2013). This measure has a high reliability for the tested age group (0.96). The Pragmatics Profile is a questionnaire containing 50 statements which the caregiver has to rate on a four-point scale. The 50 statements cover three different areas: Rituals and Conversational Skills—RCS (e.g., makes/responds to greetings to/from others), Asking for, Giving and Responding to Information—AGRI (e.g., asks for help from others appropriately), and Non-Verbal Communication—NCS (e.g., knows how someone is feeling based on non-verbal cues) (Pearson Education Inc., 2008a,b). For the rating scale, the following verbal items are used: Never, Sometimes, Often, Always. In this study the sub-scores for the three sub-measures have been used as measures in addition to the standard sum score.

#### 2.2.2. Core Language Measures

##### 2.2.2.1. Language comprehension

The Swedish version of TROG-2—Test for Reception of Grammar version 2 (Bishop, 2003; Eldblom and Sandberg, 2009), was used to assess children's language comprehension ability. This test consisted of 20 blocks of four sentences. The child saw four pictures and listened to a recorded sentence (e.g., “The star is not red”). The sentences were spoken by a native female speaker. The child was then instructed to point to the image corresponding to the sentence. The child got one point for every correct answer. After four wrong blocks in a row the test was terminated. A block was counted as being wrong if the child gave at least one wrong answer within the block. If the child did not answer at all twice in a row the test was terminated as well. In order to explain the task, two practice trials were used. The child received feedback on those two trials. The task was first continued after they gave the correct answer to both practice trials.

##### 2.2.2.2. Vocabulary skills

To test the children's vocabulary skills, the Expressive Vocabulary task from the CELF-IV battery (Semel et al., 2013) was used. This is a picture naming task. The child was shown pictures (e.g., of an elephant) and asked to name them/ a specific part of the picture (e.g., the elephant's trunk). The task started with a demonstration trial and two practice trials, after that 20 test trials followed. If the child was not able to name four pictures in a row the task was terminated. For every correctly named picture the child received one point.

#### 2.2.3. Verbal Cognitive Measures

##### 2.2.3.1. Verbal reasoning

To test verbal reasoning ability the Spoken Analogies sub-test of the Swedish ITPA-3 battery (Hammill et al., 2013) was used. The child listened to sentences of the following kind: “A dad is big, a baby is…,” and was asked to fill in the missing word. This test consisted of two practice trials and 25 test trials. The test was terminated after three consecutive incorrect answers. For every correct word, the child got one point.

##### 2.2.3.2. Verbal fluency

To test verbal fluency a semantically based fluency task was used (Benton and Hamsher, 1976). The child was asked to name as many animals as possible within 1 min. The child received one point for every animal.

##### 2.2.3.3. Verbal working memory

The sentence completion and recall task from the SIPS battery (Wass et al., 2008) was used as a measure for verbal working memory. The children heard a recorded sentence, spoken by a female speaker, with the last word missing (e.g., “A car has tires. A plane has…”). The child was then asked to fill in the missing word. A standard instruction was used and the child could practice using two examples before the real test started. There were six different levels, for level 1 children listen to two sentences, for level 2 they listen to three, for level 3 they listen to four, for level 4 they listen to two, for level 5 they listen to three, and for level 6 they listen to four sentences. The child got points for every word they recalled correctly. The test leader gave the first phoneme of the words as a cue if the child was not able to give an answer in the recall phase. If a cue was given the child only got 0.5 points for the recalled word.

### 2.3. Procedure

The Pragmatics Profile was handed out to the caregivers via the school or by the test leader and filled in at home. The rest of the testing took place at the respective school or at home. All children within the current study were tested by a speech and language pathologist or by a speech and language pathologist student in the last university term. If available in the test room a microphone and/or amplifier was used during the testing in order to enhance the speech signal for the oral test material. If these resources were not available, the child was asked if s/he wanted to use headphones to listen to the oral test material. All children preferred to use the laptop loudspeakers. The order of the tests was randomized and the test session was recorded using a Dictaphone.

### 2.4. Statistical Analysis

We used R (R Core Team, 2016) with the packages effsize (Torchiano, 2018) and cocor (Diedenhofen and Musch, 2015) for our analyses. To sort and edit the data for analysis, the packages dplyr (Wickham et al., 2019), tidyr (Wickham and Henry, 2019), and purrr (Henry and Wickham, 2019) were used. The graphs were made using the package ggplot2 (Wickham, 2009).

The alpha value was set to 0.05. All data was checked for normality and homogeneity of variance. To analyse the differences between the groups for the sum score for pragmatic language ability as well as for the sub-measure RCS, a Welch's *t*-test was used as homogeneity of variance was not given. For the other sub-measures, AGRI and NCS, a Student's *t*-test was used. To analyse the association between the language and verbal cognitive measures and pragmatic language ability, correlations have been calculated for the children without HL as well as for the children with CI. As the pragmatic measure was split into its sub-measures for the group comparison, this was also done for the correlations. For normally distributed data, Pearson correlations were calculated. For non-normally distributed data, Spearman correlations were calculated. The Benjamini-Hochberg procedure (Benjamini and Hochberg, 1995) was used to decrease the false discovery for multiple comparisons.

## 3. Results

There was no significant difference between the two groups in terms of their age, *t*_(14.41)_ = 1.00, *p* = 0.333, *d* = 0.448. Age of implantation was not significantly correlated with the pragmatic language skills of the children with cochlear implants (CI), ρ = −0.08, *p* = 0.609. Additionally, the groups did not differ in terms of their non-verbal cognitive skills [Matrix test from the Wechsler Nonverbal Scale of Ability (Wechsler and Naglieri, 2007)], *t*_(46.00)_ = 0.58, *p* = 0.567, *d* = 0.183.

### 3.1. Group Differences in Pragmatic Language Ability

The sum score of the pragmatics profile of the children without HL and the children with cochlear implants was not significantly different, *t*_(17.07)_ = 1.50, *p* = 0.152, *d* = 0.581. However 5 out of 14 children with CI had scores below the age-norm, while only 2 out of 34 children without HL performed below the age-norm. All of the children with CI who performed below the age norm attended special school. Three of them were implanted early (≤24 months), one received the implant at 36 month of age and one was implanted late (66 months).

**Figure 1:**
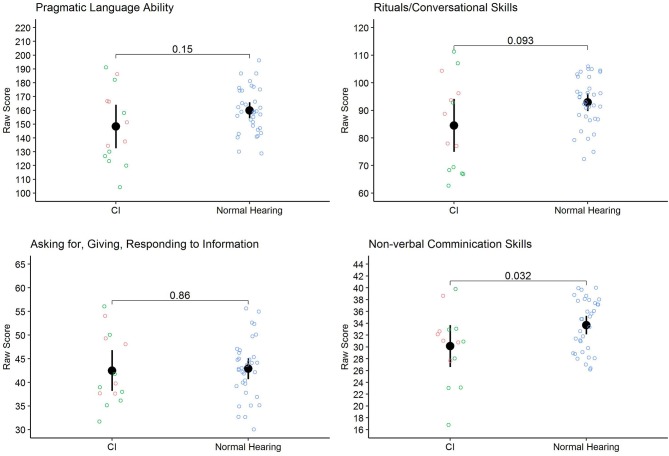
Pragmatic language skills: Raw scores for the children with CI and the children without HL. For the children with CI, green represents those attending special education and red represents those attending mainstream education.

After comparing the two groups on the sum score, sub-scores for the three measures included in the Pragmatics Profile have been calculated. For the RCS sub-measure there was no significant difference between the children without HL and the children with CI, *t*_(16.33)_ = 1.79, *p* = 0.093, *d* = 0.717. For the AGRI sub-measure no significant difference was found between the groups, *t*_(46.00)_ = 0.18, *p* = 0.858, *d* = 0.057. For the NCS sub-measure a significant difference, *t*_(46.00)_ = 2.22, *p* = 0.032, *d* = 0.704, was found between the groups with children without HL performing better than the children with CI. This difference was still significant when excluding the two items “using a variation of tone of voice” and “recognizing that other people use different tone of voice” which could be argued are influenced by hearing with a CI, *t*_(46.00)_ = 2.19, *p* = 0.033, *d* = 0.696). For a graphical representation of the results (see Figure 1), means, standard deviation, and range are reported in Table 2.

**Table 2:** Descriptive data for the pragmatic language skills of children with CI and children without HL.

	**Pragmatic profile sum score**			**RCS**			**AGRI**			**NCS**		
	***M***	**SD**	**Range**	***M***	**SD**	**Range**	***M***	**SD**	**Range**	***M***	**SD**	**Range**
Children with CI	148.29	27.27	[104, 191]	84.5	16.62	[63, 111]	42.5	7.5	[32, 56]	30.14	6.14	[17, 40]
Children without HL	160	16.51	[129, 196]	92.91	9.12	[72, 106]	42.88	6.32	[30, 56]	33.67	4.5	[26, 40]

### 3.2. Association Between Language and Verbal Cognitive Measures and Pragmatic Language Ability

#### 3.2.1. Children With CI

All three pragmatic sub-measures: RCS, ρ = 0.64, *p* = 0.040, AGRI, ρ = 0.74, *p* = 0.021, and NCS, ρ = 0.66, *p* = 0.040, were significantly positively correlated with verbal fluency but no other measure.

#### 3.2.2. Children Without HL

The RCS score of the children without HL was significantly positively correlated with their language comprehension ability, *r* = 0.40, *p* = 0.033, and their verbal reasoning ability, ρ = 0.46, *p* = 0.017. The AGRI score of the children without HL was not significantly correlated with any of the measured skills. The NCS score of the children without HL was significantly positively correlated with their vocabulary skill, ρ = 0.49, *p* = 0.013, and with their verbal reasoning ability, ρ = 0.53, *p* = 0.009.

## 4. Discussion

The aim of the current study was to analyse whether there is a difference between children using CI and children without HL in terms of their pragmatic language ability. In addition associations between pragmatic language ability and different verbal cognitive and language measures were analyzed, first to see which skills are possibly influencing the pragmatic language ability of children with CI and second to analyse differences in relationship patterns of children with CI and children without HL.

No significant difference was found between the children without HL and the children with CI for the sum score of the pragmatics language measure. This is in accordance with the results found by Guerzoni et al. (2016). It differs, however, from results suggesting differences between children with CI and children without HL in terms of their pragmatic language ability (Jeanes, 2000; Most et al., 2010; Goberis et al., 2012; Rinaldi et al., 2013). The present study, as well as the study by Guerzoni et al. (2016), used parent rating, while in other studies the researches have rated the conversational skills of the children while communicating with a familiar adult (Most et al., 2010) or a peer (Jeanes, 2000; Ibertsson et al., 2009; Toe and Paatsch, 2013). It could be argued that parents tend to overestimate the competence of their child. However, the reliability of the measure used in the present study has been reported to be high (*r*^a^ = 0.96) (Semel et al., 2013). In addition, other studies (Goberis et al., 2012; Rinaldi et al., 2013) have found poorer ratings for pragmatic language ability for deaf and hard of hearing children compared to children without HL even when being judged by their parents. Certainly, as the use of different measures of pragmatic language ability as well as different age groups, make it hard to directly compare results between studies. Different pragmatic language measures often include different domains of pragmatic language ability (Russell and Grizzle, 2008), which means that even if differences have been found in one specific measure, this does not necessarily mean that the two groups differ on all aspects of pragmatic language ability. In addition, although no significant differences could be seen on group level, 5 out of 14 children with CI (35.71%) performed below the age norm, while only 2 out of 34 children without HL (5.89%) performed below the age norm. All children with CI performing below the age-norm were attending special school. This is in accordance with the results of Thagard et al. (2011), who found a correlation between time spent in general education and pragmatic language ability. However, for both the results of the current study and the results found by Thagard et al. (2011) it is unclear if children having problems in the pragmatic language domain are the ones in need of special education or if being in special education leads to a delay in pragmatic language skills. Most et al. (2010) argue that one reason for the poorer pragmatic language ability of deaf and hard of hearing children might be that they have fewer possibilities to practice. This might especially be the case for children attending special education as they may have even fewer possibilities to engage in discourse with hearing peers or hearing adults who are not trained to talk to deaf and hard of hearing children, or to use sign support in comparison to children with CI attending mainstream education. Further studies should evaluate whether and how communication behavior in school and at home influences the pragmatic language ability of children with CI. Increased knowledge about this topic would benefit the development of intervention programs to improve the development of pragmatic language.

In the present study a significant difference between children with CI and children without HL was found for the Nonverbal Communication skills (NCS) sub-measure. Intuitively, non-verbal communication skills should not be influenced by having a hearing loss. In addition, Most et al. (2010) found no difference between children without HL and deaf and hard of hearing children neither on a non-verbal communication nor on a para-linguistic scale for pragmatic language ability. This is therefore a surprising result. Two items included in the NCS measure are: “varying tone of voice” and “recognizing varying tone of voice.” It could be argued that those two skills are influenced by hearing with a CI. Comparing the two groups on the NCS scale without including those two items, however, still led to a significant group difference. This means hearing ability does not seem to be the main issue. A number of the items used in the sub-measure NCS for example “being able to recognize how somebody is feeling” or “understanding facial expressions” could be related to Theory of Mind development. The term “Theory of Mind” (ToM) refers to the ability to know about your own and other people's mental states. A child who can attribute beliefs, knowledge, emotions, desires, and intentions to other people and understands that those may differ from his/her own beliefs, knowledge, emotions, desires, and intentions has mastered ToM. This is usually the case around age five to six (Liu et al., 2018). A child with fully developed ToM skills should be able to recognize how somebody is feeling as well as understand facial expressions. Even the ability to recognize varying tone of voice is important, as distinct emotional states or intentions might be indicated by differences in tone of voice. Children who have fully developed ToM skills should therefore have higher scores on the NCS sub-measure. Studies have found that the development of ToM is often delayed in deaf and hard of hearing children (Peterson and Siegal, 2000; Lundy, 2002; Peterson, 2004; Ketelaar et al., 2012; Liu et al., 2018). In a meta-analysis done by Milligan et al. (2007), significant relations between language ability and ToM have been found. As children with CI are often delayed in terms of their language development, their delayed development of ToM is no surprise. Peterson (2004) found that children with CI perform on par with age-matched children with autism on tasks measuring ToM. The authors argue that restricted discourse between deaf and hard of hearing children and their hearing parents could be a reason for the delayed development. This is in accordance with the suggestion by Most et al. (2010) that pragmatic language development could be influenced by the opportunities to practice conversations.

No significant group difference was found for the Rituals and Conversational skills (RCS) and Asking for, Giving, and Responding to Information (AGRI) sub-measures. This is a promising result. Children with CI seem to be able to master these important parts of pragmatic language ability. For the AGRI sub-measure the result is in accordance with previous studies. This measure involves the abilities to ask for clarification, reacting to requests for clarification, explaining, and asking why things are like they are and why people do what they do, as well as a number of social skills, like asking for help, accepting apologies etc. Jeanes (2000) found that deaf and hard of hearing children using oral language used even more requests for clarifications than did children without HL and that they responded appropriately to requests for clarification. Furthermore, Antia et al. (2011) reported that the social skills of deaf and hard of hearing children are within their age norm. For the RCS sub-measure, the results are in accordance with studies suggesting that children with CI have conversational skills that are good enough to ensure a fluent conversation with a hearing peer (Toe and Paatsch, 2013). It differs, however, from other results suggesting difficulties of children with CI with verbal turn taking (Most et al., 2010; Paatsch and Toe, 2014). It should be mentioned that although the difference for the RCS sub-measure was not significant, there was a tendency for the children without HL to obtain higher scores than the children with CI, and the accompanying effect size was as high as it was for the NCS sub-measure. As the sub-measure included not only conversational skills but also the use of rituals, like saying hello or goodbye, it might be the case that some but not all of the abilities measured differed between the groups.

The correlation patterns between pragmatic language ability and language and verbal cognitive ability was different for children with CI and the children without HL. For the children without HL language comprehension as well as verbal reasoning were positively correlated with the RCS scale. Furthermore, vocabulary skills and verbal reasoning were positively correlated with the NCS scale. As Matthews et al. (2018) point out it is often hard to distinguish between language skills, like language comprehension and vocabulary skills, and pragmatic language ability, a correlation between those skills was therefore expected. In addition the significant correlation between pragmatic language skills and verbal reasoning is in accordance to results from a study by Turkstra et al. (1996). Turkstra et al. (1996) suggest that inferential reasoning is important for pragmatic language ability and these two abilities are therefore associated.

For the children with CI, verbal fluency was the only skill correlated with all three sub-measures of pragmatic language ability. Previous studies (Kenett et al., 2013; Wechsler-Kashi et al., 2014) found that children with CI have a less developed semantic network. Semantic network here refers to the organization of words and different word meanings within the mental lexicon. Wechsler-Kashi et al. (2014) evaluated the organization of the semantic network of children with CI using verbal fluency tasks. The authors suggest that the children performed more poorly than children without HL as lexical organization is underdeveloped. The results from a computational analysis done by Kenett et al. (2013) support this view. Children with CI seem to have less strong connections between different words. Because of that, the activation of one word in their semantic network does not spread as much as it does for children without HL. The better the semantic network is developed, the better the performance on a verbal fluency task as more words are activated and their retrieval is therefore eased. The results from the current study suggest that children with CI who have a better developed semantic network have higher pragmatic language ability. A reason for these findings might be that a more structured network enables language to be used in a more flexible way. However, as only correlations have been used in the current study the causal direction is not clear. It could be that the quality and quantity of face-to-face interactions influence both the structure of the semantic network as well as pragmatic language ability. In addition, no correlations between verbal fluency and pragmatic language ability have been found for the children without HL. It might be that children with CI use different strategies for social communication that are more influenced by their semantic network. It might also be that the semantic network of children without HL is developed to a degree where more improvement does not influence pragmatic language ability anymore. More studies are needed to untangle the relationship pattern between hearing loss, verbal fluency, and pragmatic language ability.

### 4.1. Limitation of the Study

In the present study a small sample of children with CI was tested. It is possible that significant differences were therefore not detected for some of the variables. There was a tendency for a difference on the RCS measure and the accompanying effect size was fairly high. It is likely that a large sample size would have been needed to detect the significant difference of the groups on the RCS scale. In the present study we have used a parent rating to measure pragmatic language skills. While this offers the possibility to get more insight into real-life pragmatic language skill compared to when analyzing conversations in the lab it also leads to some disadvantages. First it is a subjective measure. Further studies should aim to combine subjective and objective measures to get a better insight into the pragmatic language skills of children with CI. Second it is an inclusive measure. This makes it possible to get a broad overview over the current status of pragmatic language skill development but makes it hard to analyse which specific sub-skill might be causing differences. Differences between sub-skills might even go unnoticed if they are only measured by one or two items and differences therefore don't lead to significant differences on the sum measure or on the sub-measure level. Further research should aim to get data for a bigger group of children to be able to do a more precise item analysis to evaluate differences on a more detailed level. A further limitation of the study is the heterogeneity in terms of age of implantation of the children with CI. However, age of implantation was not correlated with pragmatic language skills and removing the two children with the oldest implantation age (60 and 66 months, leading to a SD of 11 months) did not change the results. Further studies should aim to collect more data concerning the pragmatic language skill of children with CI to be able to analyze the influence of age of implantation in more detail. A further limitation of the study is the missing information about the pre-implant hearing thresholds of the children. It might be the case that the degree of hearing loss influences the pragmatic language development. It is important to conduct more research on this topic to evaluate which other factors apart from verbal fluency are of importance for the pragmatic language development of children with CI. One additional factor might be the socioeconomic status of the parents. Rowe (2008) has reported a relation between child-directed speech and socioeconomic status of the parents. As child-directed speech is likely to influence pragmatic language development it is important for further studies to take the influence of this variable into consideration.

## 5. Conclusion

The results of the current study suggest that many children with CI show pragmatic language ability comparable to their hearing peers and in accordance to their age-norm. In the present study, significant differences were found on a measure connected to theory of mind, a skill found to be delayed in deaf and hard of hearing children. It has been suggested that the quality and quantity of face-to-face interactions influence both theory of mind and pragmatic language ability. Further studies are needed to analyse the influence of communication styles of care givers, teachers and peers on the development of pragmatic language ability in children. Results from the current study show that the development of the semantic network is associated with pragmatic language ability of children with CI. Verbal fluency was correlated with all three sub-measures of pragmatic language ability. The causal direction is unclear. It might be that children with a better developed semantic network are able to use language in a more flexible way. Alternatively, quality and quantity of oral interaction might influence both the development of the semantic network and of pragmatic language ability. To be able to develop interventions for children with CI showing problems in the pragmatic language domain it is important to get more insight into the connection between conversation, verbal fluency, and pragmatic language ability.

## Data Availability Statement

The datasets generated for this study will not be made publicly available. It was ensured to the parents in the information letter that no data will be send to anyone not part of the research team. This was also included in the ethics application.

## Ethics Statement

The studies involving human participants were reviewed and approved by Regionala etikprövningsnämnden i Linköping. Written informed consent to participate in this study was provided by the participants' legal guardian/next of kin.

## Author Contributions

The study was prepared and designed by MS, BL, MG, IH, and MW. Acquisition of data was done by MG, IH together with Linn Hellgren and Elias Larsson (acknowledged). Analysis and interpretation of result was carried out by mainly MS, RE, and MW. The first draft of the manuscript was written by MS. All authors took part in critical revision of the manuscript.

### Conflict of Interest

The authors declare that the research was conducted in the absence of any commercial or financial relationships that could be construed as a potential conflict of interest.
